# A Single-Cell Transcriptional Landscape of Emphysema: Heterogeneous Cellular Dynamics in Centrilobular, Panlobular, and Paraseptal Subtypes

**DOI:** 10.3390/ijms27177807

**Published:** 2026-08-31

**Authors:** Wei-Ping Hu, Jian-Cheng Zhu, Li Liu, Yi-Xing Wu, Jie Wang, Jing Zhang

**Affiliations:** 1 Department of Pulmonary and Critical Care Medicine, Zhongshan Hospital, Shanghai Medical College, Fudan University, Shanghai 200032, China; wphu@foxmail.com (W.-P.H.); 20301050230@fudan.edu.cn (J.-C.Z.); llzero9@163.com (L.L.); wuyixing30@163.com (Y.-X.W.); 2 Department of Thoracic Surgery and State Key Laboratory of Genetic Engineering, Fudan University Shanghai Cancer Center, Shanghai 200032, China

**Keywords:** emphysema, centrilobular emphysema, panlobular emphysema, paraseptal emphysema, single-cell RNA sequencing

## Abstract

Emphysema is a chronic lung disease characterized by irreversible alveolar destruction, with distinct imaging subtypes—centrilobular emphysema (CLE), panlobular emphysema (PLE), and paraseptal emphysema (PSE). The cellular and molecular mechanisms driving subtype-specific pathogenesis remain poorly understood. Here, we performed single-cell RNA sequencing on emphysematous lung tissues from nine patients (three per subtype) to map the transcriptional landscape across CLE, PLE, and PSE. Profiling 84,704 high-quality cells, we identified eight major cell types, and subsequent subclustering uncovered subtype-enriched populations and transcriptional features. CLE was characterized by upregulated inflammatory pathways in ciliated cells and activated macrophage–epithelial/fibroblast crosstalk, suggesting an airway-derived infection and inflammatory phenotype. In PLE, the proportions of Natural Killer T (NKT) cells and Group 1 innate lymphoid cells (ILC1s) with enhanced cytotoxicity were increased, and mesothelial cells showed upregulation of extracellular matrix (ECM)-related genes, indicating dysfunction in innate immune cells and abnormal repair of mesothelial cells. PSE showed pronounced ECM remodeling signatures, with an increased proportion of systemic venous endothelial cells and enhanced fibroblast–epithelial interactions. Collectively, our findings reveal that CLE is predominantly driven by airway-originated inflammation, PSE exhibits a fibrosis-like remodeling phenotype, and PLE represents an intermediate state of innate immune activation and matrix dysregulation, providing a single-cell resolution framework for subtype-specific therapeutic targeting.

## 1. Introduction

Emphysema is characterized by abnormal permanent enlargement of the air space distal to the terminal bronchioles, accompanied by destruction of the alveolar walls [1]. As a common chronic lung disease, the prevalence of emphysema varies across different populations, mainly influenced by smoking behavior and the prevalence of chronic obstructive pulmonary disease (COPD) [2,3,4]. In the general population, the detection rate of emphysema is approximately 8.7%, while it increases significantly to over 30% in individuals with airflow limitation [5]. The structural damage leads to loss of elastic recoil, reduced gas-exchange surface, and increased lung compliance, resulting in progressive dyspnea and impaired exercise capacity [6]. Multiple factors, including cigarette exposure, air pollution, genetic susceptibility, and chronic airway inflammation, contribute to the development of emphysema [7,8,9]. At the molecular level, the mechanisms include protease/antiprotease imbalance, increased oxidative stress, dysregulation of fundamental cellular processes (e.g., programmed cell death and autophagy), and impaired tissue repair [10]. The alveolar structural damage in emphysema is irreversible, and effective pharmacological treatments remain limited. Therefore, further elucidation of the underlying molecular mechanisms is needed.

Emphysema is categorized into several subtypes based on the anatomic location of alveolar destruction, including centrilobular emphysema (CLE), panlobular emphysema (PLE), and paraseptal emphysema (PSE) [11]. CLE is characterized by enlargement of airspaces in the center of the secondary lobule, whereas distal alveolar ducts and sacs remain normal. PLE is defined by homogeneous dilation of airspaces from the respiratory bronchioles to the alveoli, with uniform changes throughout the secondary lobule. PSE affects pulmonary lobules adjacent to the pleural surface and predisposes to pneumothorax.

Studies on emphysema subtypes have predominantly focused on imaging-based assessment and the clinical prognosis of patients [12,13,14]. Only a few studies have investigated differences in immune cell infiltration, suggesting significantly higher infiltration of neutrophils and fewer mast cells in PSE than in CLE [15,16]. However, mechanistic investigations aimed at elucidating the molecular and cellular pathways underlying different emphysema subtypes remain limited.

Single-cell RNA (scRNA) sequencing enables gene expression profiling at the resolution of individual cells, which can reveal cellular heterogeneity and specific molecular changes. Recent scRNA sequencing studies of lung tissues from patients with COPD have revealed cell-specific mechanisms and highlighted the critical role of immune dysregulation in the small airway regions in the pathogenesis and progression of COPD [17,18]. However, scRNA sequencing studies focusing on the heterogeneity among emphysema subtypes are still lacking. In this study, we utilized scRNA sequencing analysis to systematically dissect the cellular landscape and molecular characteristics of various emphysema subtypes. By profiling individual cells at high resolution, we sought to uncover subtype-specific alterations in gene expression, intercellular communication networks, and key regulatory pathways that could provide novel insights into the pathophysiology of emphysema.

## 2. Results

### 2.1. Cellular Heterogeneity and Global Transcriptomic Signatures Across Three Emphysema Subtypes

**Figure 1:**
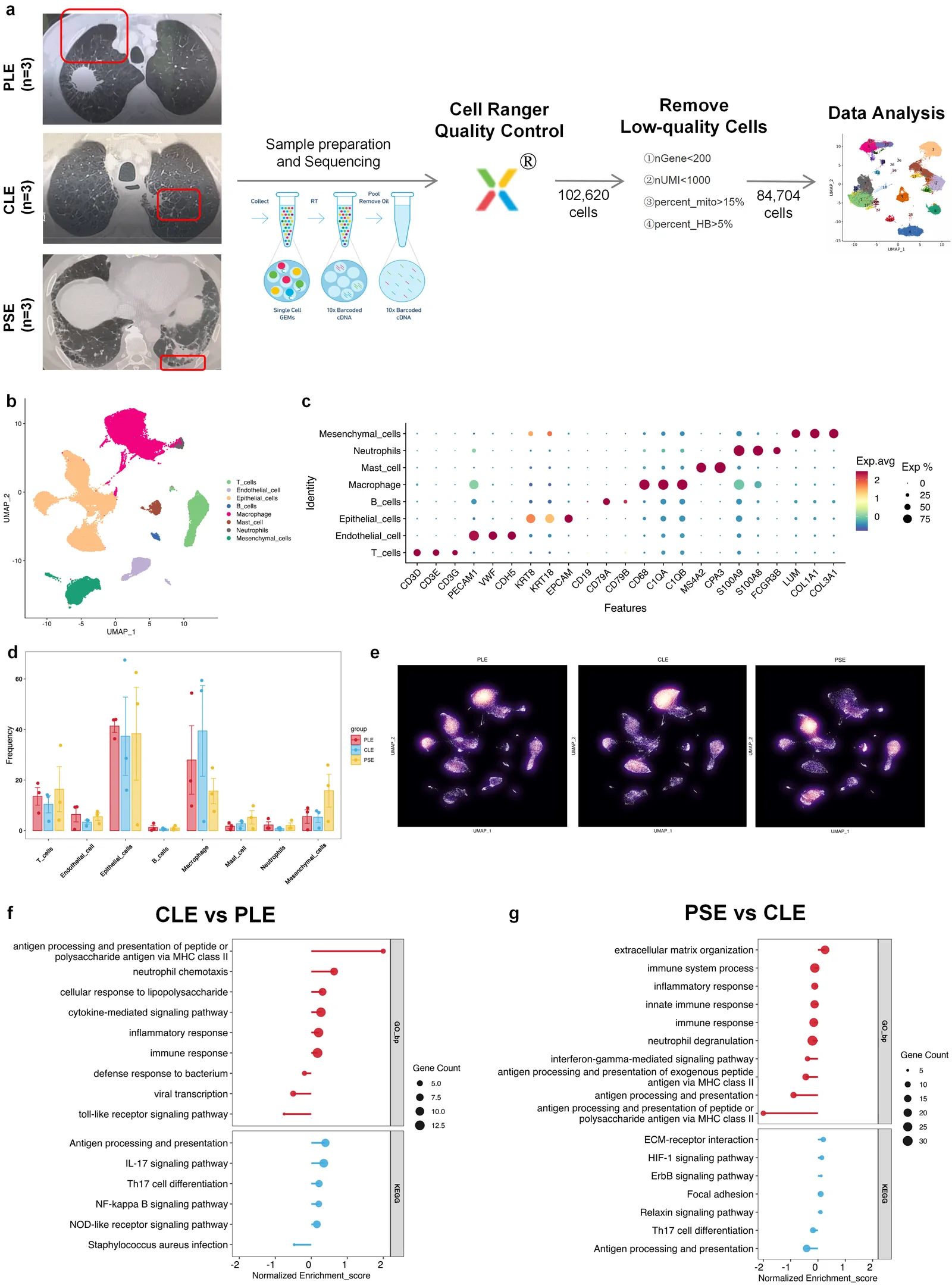
Distinct cellular abundance and transcriptomic features among three emphysema subtypes. (**a**) Representative Computed tomography (CT) images of each emphysema subtype and the subsequent workflow. The areas highlighted by the red boxes show the manifestations of different emphysema subtypes in CT images. (**b**) UMAP plot shows the 8 major distinct cellular clusters. (**c**) The expression patterns of lineage markers across different cell types. (**d**,**e**) Bar plot and density plot show the relative proportions of each cell type across the three emphysema subtypes, respectively.The brighter areas indicate a higher number of cells (**f**) Bubble plot shows the GO_BP and KEGG enrichment analysis of DEGs between CLE and PLE. (**g**) Bubble plot shows the GO_BP and KEGG enrichment analysis of DEGs between PSE and CLE. Abbreviations: CLE, centrilobular emphysema; PLE, panlobular emphysema; PSE, paraseptal emphysema; UMAP, uniform manifold approximation and projection; GO_BP, Gene Ontology biological process; KEGG, Kyoto Encyclopedia of Genes and Genomes; DEGs, differentially expressed genes.

As illustrated in Figure 1a, we conducted scRNA-seq analysis on nine samples of emphysematous tissue adjacent to tumors, obtained from patients undergoing surgical resection of lung tumors. As summarized in Table 1, the demographic and clinical characteristics of the nine participants were balanced across the three emphysema subtypes.

After rigorous quality control, we included 84,704 high-quality cells for downstream analyses (Figure S1). After normalizing the gene expression, we performed principal components analysis (PCA) on the top 2000 most variably expressed genes across all cell samples, selecting 21 principal components (PCs) for uniform manifold approximation and projection (UMAP).

Based on canonical lineage markers, we identified eight major distinct cellular clusters, including epithelial cells, endothelial cells, mesenchymal cells, macrophage cells, T cells, B cells, neutrophils and mast cells (Figure 1b,c). As shown in Figure 1d,e, CLE and PLE had a higher frequency of macrophages compared with PSE. In addition, PSE showed a higher frequency of mesenchymal cells and mast cells than CLE and PLE. However, these differences did not reach statistical significance. The proportions of each cellular cluster across the three groups and nine samples were supplemented in Figure S2.

In Tables S1 and S2, we performed global differential gene expression and pathway enrichment analyses on the overall cellular profiles, comparing each pair of emphysema subtypes: ① between CLE and PLE, and ② between PSE and CLE. In the comparison between CLE and PLE, we found that genes significantly upregulated in CLE were enriched in antigen processing and presentation, cytokine-mediated signaling pathway, inflammatory response and immune response (Figure 1f). In the comparison between PSE and CLE, genes significantly upregulated in PSE were enriched in extracellular matrix (ECM) organization and ECM–receptor interaction (Figure 1g).

Following unbiased clustering and removal of cells with ambiguous marker profiles, six major cellular clusters were further subdivided into several subclusters. Lineage markers used for cluster annotation are provided in Table S3. Top 10 marker genes and Gene Set Variation Analysis (GSVA)-based functional annotations were presented in Tables S3 and S4. The proportional differences in each subcluster across the three emphysema subtypes were shown in the stacked bar plots in Figure S3. The results of pseudotime trajectory analyses for subclusters were provided in Figures S4 and S5.

### 2.2. CLE: Upregulated Airway-Derived Infection and Inflammatory Pathways

#### 2.2.1. Ciliated Epithelium in CLE Exhibits an Upregulated Inflammatory Phenotype

**Figure 2:**
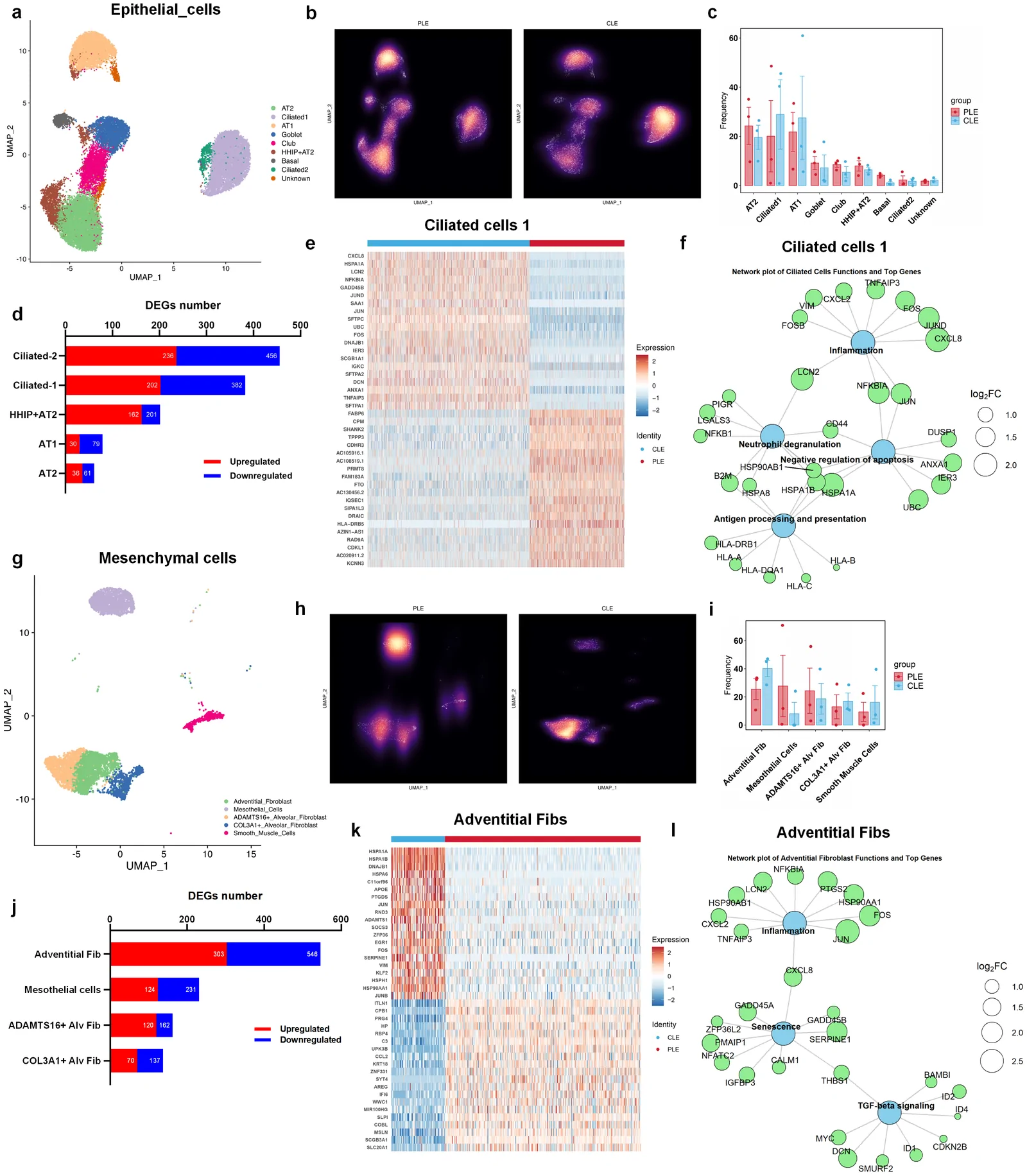
Distinct abundance and transcriptional traits of ciliated cells and adventitial fibroblasts in CLE. (**a**) UMAP plot shows nine distinct subclusters of epithelial cells. (**b**,**c**) Density plot and bar plot show the relative proportions of epithelial cell subtypes in comparison between PLE and CLE, respectively.The brighter areas indicate a higher number of cells. (**d**) Bar plot shows the number of upregulated and downregulated DEGs in each epithelial cell subtype between CLE and PLE. (**e**) Heatmap shows the top 20 DEGs in Ciliated cells #1 between CLE and PLE. (**f**) Network plot illustrates enriched functional modules (blue nodes) and associated genes (green nodes) in Ciliated cells #1. The size of green nodes corresponds to the magnitude of differential expression (log2 fold change) between CLE and PLE. (**g**) UMAP plot shows five distinct subclusters of mesenchymal cells. (**h**,**i**) Density plot and bar plot show the relative proportions of mesenchymal cell subtypes in comparison between PLE and CLE, respectively. The brighter areas in (**h**) indicate a higher number of cells. (**j**) Bar plot shows the numbers of upregulated and downregulated DEGs in each mesenchymal subtype between CLE and PLE. (**k**) Heatmap shows the top 20 DEGs in adventitial fibroblasts between CLE and PLE. (**l**) Network plot illustrates enriched functional modules (blue nodes) and associated genes (green nodes) in adventitial fibroblasts. Abbreviations: CLE, centrilobular emphysema; PLE, panlobular emphysema; UMAP, uniform manifold approximation and projection; DEGs, differentially expressed genes.

Epithelial cells were further subjected to dimensionality reduction and unsupervised clustering, resulting in nine distinct clusters. Based on their expression profiles, these clusters were annotated as Alveolar type 2 epithelial cells (AT2), Ciliated cells #1, Alveolar type 1 epithelial cells (AT1), Goblet cells, club cells, *HHIP*^+^ AT2, basal cells, Ciliated cells #2, and an unknown population (Figure 2a). Comparative analysis revealed that the proportion of AT2 cells was lower in CLE than in PLE, whereas Ciliated cells #1 were enriched in CLE, though these differences did not reach statistical significance (Figure 2b,c). DEGs analysis demonstrated that genes upregulated in CLE compared with PLE were predominantly concentrated in Ciliated cells #1 and #2 (Figure 2d), suggesting a potentially important role of ciliated cells in CLE. The heatmap of the top 20 differentially expressed genes (DEGs) revealed that several inflammation-associated genes were upregulated in Ciliated cells #1, including *CXCL8*, *NFKBIA*, *FOS*, *JUND*, etc. (Figure 2e). Functional enrichment analysis of the upregulated genes in CLE ciliated cells showed that these genes were mainly associated with inflammation, neutrophil degranulation, negative regulation of apoptosis, and antigen processing and presentation (Figure 2f). In addition, the top 20 DEGs of all subclusters and significantly enriched pathways (CLE vs. PLE) are listed in Tables S5 and S6.

#### 2.2.2. Adventitial Fibroblasts in CLE Exhibit Inflammatory and Senescent Phenotypes

Mesenchymal cells were further subjected to dimensionality reduction and unsupervised clustering, resulting in five distinct clusters. Based on their expression profiles, these clusters were annotated as adventitial fibroblasts, mesothelial cells, *ADAMTS16*^+^ alveolar fibroblasts, *COL3A1*^+^ alveolar fibroblasts, and smooth muscle cells (Figure 2g). Comparative analysis showed that the proportion of mesothelial cells was reduced in CLE compared with PLE, whereas adventitial fibroblasts were enriched in CLE (Figure 2h,i). Analysis of DEGs revealed that genes upregulated in CLE relative to PLE were predominantly concentrated in adventitial fibroblasts (Figure 2j). The heatmap showed the top 20 DEGs in adventitial fibroblasts, including *ADAMTS1*, *SOCS3*, *VIM* and *KLF2*. Functional enrichment analysis of the upregulated genes in CLE adventitial fibroblasts indicated that these genes were mainly associated with inflammation, senescence, and Transforming growth factor-β (TGF-β) signaling (Figure 2i).

#### 2.2.3. All Macrophage Subtypes in CLE Were Activated and Exhibited Enhanced Interactions with Tissue Cells

**Figure 3:**
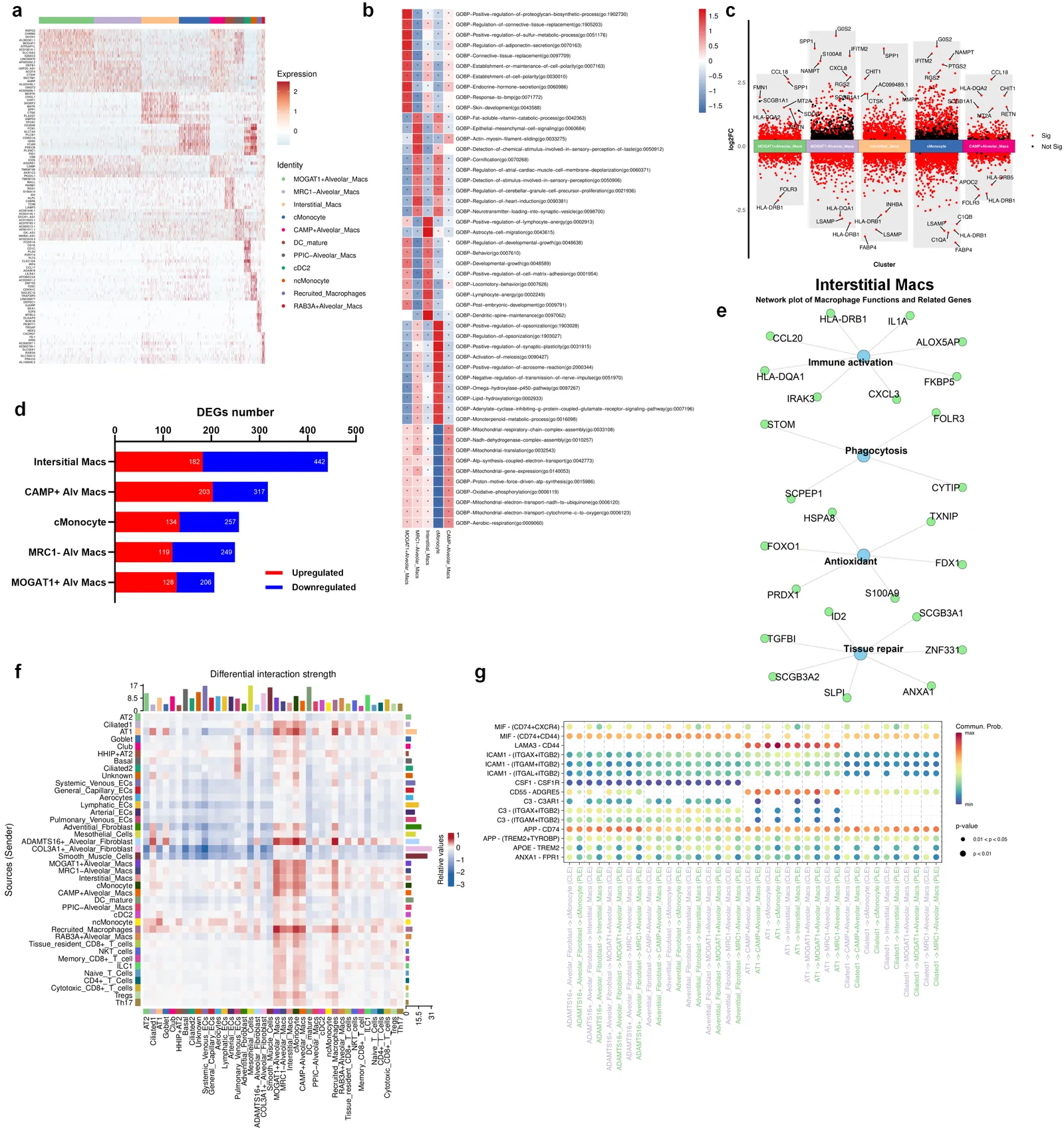
Distinct abundance and transcriptional traits of macrophages in CLE. (**a**) Heatmap shows the expression of the top 10 marker genes in each macrophage subcluster. (**b**) Heatmap shows GSVA differences in GO_BP terms across the top 5 macrophage subclusters. * indicates that the *p*-value for the term in the corresponding group is <0.05 (**c**) Multi-volcano plot shows the upregulated and downregulated DEGs in the top 5 macrophage subclusters between CLE and PLE. (**d**) Bar plot shows the numbers of upregulated and downregulated DEGs in each macrophage subcluster between CLE and PLE. (**e**) Network plot illustrates enriched functional modules (blue nodes) and associated genes (green nodes) in interstitial macrophages. (**f**) Heatmap shows differences in inferred cell–cell interaction strength among epithelial cells, endothelial cells, macrophages, mesenchymal cells, and T cells between CLE and PLE. (**g**) Dot plot shows inferred cell–cell communication probabilities between ligand–receptor pairs across major cell types from CLE and PLE. Abbreviations: CLE, centrilobular emphysema; PLE, panlobular emphysema; GSVA, Gene Set Variation Analysis; GO_BP, Gene Ontology biological process; DEGs, differentially expressed genes.

Macrophages were further subjected to dimensionality reduction and unsupervised clustering into eleven subclusters, and their top 10 marker genes are shown in Figure 3a. The top five subclusters were named as *MOGAT1*^+^ alveolar macrophages, *MRC1*^−^ alveolar macrophages, interstitial macrophages, cMonocyte and *CAMP*^+^ alveolar macrophages. GSVA revealed distinct molecular functions and enriched pathways among these subtypes. Specifically, *MOGAT1*^+^ alveolar macrophages were associated with ECM production, TGF-β signaling, and lipid-related processes. *MRC1*^−^ alveolar macrophages were enriched in epithelial–mesenchymal transition signaling and cytoskeletal regulation. Interstitial macrophages were primarily involved in lymphocyte-related processes. *CAMP*^+^ alveolar macrophages were characterized by mitochondrial function and oxidative stress-related pathways (Figure 3b). However, these five macrophage subtypes shared a similar set of DEGs, such as *SPP1*, *CCL18*, *SCGB1A1*, and *NAMPT* (Figure 3c,d). In the comparison of CLE and PLE, interstitial macrophages displayed 182 upregulated and 260 downregulated genes (Figure 3d), which were enriched in immune activation, phagocytosis, antioxidant and tissue repair (Figure 3e). Among these genes, *FKBP5*, a gene that negatively regulates glucocorticoid sensitivity, was significantly upregulated in CLE.

Cell–cell communication analysis suggested that, compared with PLE, macrophage interactions with epithelial and fibroblast cells were significantly enhanced in CLE. This effect was particularly evident in *MOGAT1*^+^ alveolar macrophages, cMonocytes, ciliated cells #1, AT1 cells, adventitial fibroblasts, and *ADAMTS16*^+^ alveolar fibroblasts (Figure 3f). Among ligand–receptor pairs, the MIF–(CD74-CXCR4) axis showed the most pronounced difference, followed by the APOE-TREM2 and ANXA1-FPR1 interactions (Figure 3g).

### 2.3. PLE: Innate Immune Activation and Increase in Mesothelial Cells

#### 2.3.1. Increased Proportion of Natural Killer T (NKT) Cells and Group 1 Innate Lymphoid Cells (ILC1s) in PLE

**Figure 4:**
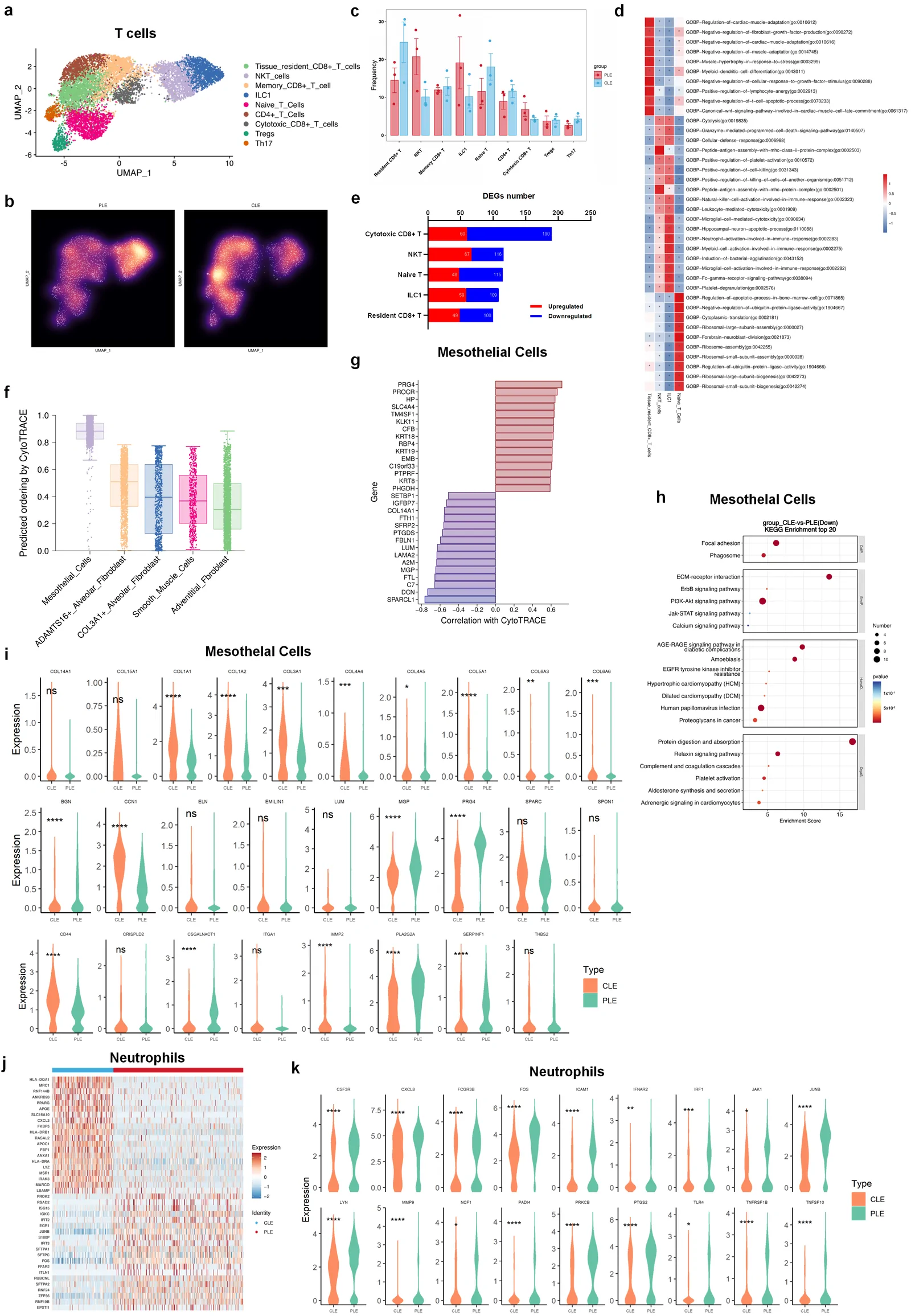
Distinct abundance and transcriptional traits of T cells, mesothelial cells, and neutrophils in PLE. (**a**) UMAP plot shows nine distinct subclusters of T cells. (**b**,**c**) Density plot and bar plot show the relative proportions of T cell subtypes in PLE and CLE, respectively. The brighter areas in (**b**) indicate a higher number of cells. (**d**) Heatmap shows GSVA differences in GO_BP terms across the top 5 T cell subclusters. * indicates that the *p*-value for the term in the corresponding group is <0.05 (**e**) Bar plot shows the number of upregulated and downregulated DEGs in each T cell subtype between CLE and PLE. (**f**) Box plot shows the predicted differentiation potential of mesenchymal cell subtypes based on CytoTRACE analysis. (**g**) Bar plot shows genes in mesothelial cells with high correlation to CytoTRACE scores. (**h**) Bubble plot shows enriched KEGG pathways of mesothelial cells between CLE and PLE. (**i**) Violin plot shows the expression levels of ECM-related genes of mesothelial cells between CLE and PLE. (**j**) Heatmap shows the expression patterns of the top 20 DEGs in neutrophils between CLE and PLE. (**k**) Violin plot shows the expression levels of genes related to neutrophils’ core functions between CLE and PLE. Abbreviations: CLE, centrilobular emphysema; PLE, panlobular emphysema; UMAP, uniform manifold approximation and projection; GSVA, Gene Set Variation Analysis; GO_BP, Gene Ontology biological process; KEGG, Kyoto Encyclopedia of Genes and Genomes; DEGs, differentially expressed genes; ECM, extracellular matrix. *p* < 0.05 (*), *p* < 0.01 (**), *p* < 0.001 (***), *p* < 0.0001 (****), ns, no significance.

T cells were further subjected to dimensionality reduction and unsupervised clustering, resulting in nine distinct clusters (Figure 4a). Based on their expression profiles, these clusters were annotated as Tissue-resident CD8+ T cells, NKT cells, Memory CD8+ T cells, ILC1, Naive T cells, CD4+ T cells, Cytotoxic CD8+ T cells, Tregs and Th17 cells (Figure 4a). Comparative analysis showed that the proportions of NKT cells and ILC1 were higher in PLE compared with CLE, whereas the proportions of Tissue-resident CD8+ cells and Naive T cells were decreased in PLE (Figure 4b,c). GSVA was subsequently performed to characterize functional traits of T-cell subsets. NKT cells displayed enrichment of cytotoxicity-related programs, including cell killing, cytolysis, granzyme-mediated programmed cell death, and natural killer cell-mediated immune responses. Similarly, ILC1 cells were characterized by enrichment of cytolytic and degranulation pathways, as well as immune effector functions involving leukocyte, neutrophil, and myeloid cell activation (Figure 4d).

#### 2.3.2. Mesothelial Cells Were Increased and Characterized by Upregulated ECM-Related Genes in PLE

Figure 2h,i showed that mesothelial cells were markedly increased in PLE. Cell trajectory reconstruction analysis using gene counts (CytoTRACE) analyses suggested that, compared with other mesenchymal cells, mesothelial cells displayed the lowest differentiation state and retained differentiation potential. Notably, *PRG4* showed the strongest association with this low differentiation state (Figure 4f,g). Enrichment analyses suggested that, compared with CLE, pathways including ECM–receptor interaction, focal adhesion, erythroblastic leukemia viral oncogene homolog (ErbB) signaling pathway, Phosphatidylinositol 3-kinase–Akt (PI3K-Akt) signaling pathway and protein digestion and absorption were upregulated in mesothelial cells from PLE (Figure 4h). The violin plots showed the significantly differentially expressed ECM-related genes in mesothelial cells, including *PRG4*, *PLA2G2A*, *CD44*, *CCN1*, *COL1A1*, and *COL1A2*.

#### 2.3.3. Neutrophils in PLE Display Obvious Transcriptional Perturbations

Figure 1d,e shows that neutrophils are increased in PLE compared with CLE. The heatmap showed the top 20 DEGs of neutrophils, and several genes related to neutrophil’s core functions were significantly upregulated in PLE (Figure 4j,k), including migration and chemotaxis (*CSF3R*, *ICAM1*, *CXCL8*), pathogen recognition and phagocytosis (*TLR4*, *FCGR3B*), cytotoxicity and degradation (*NCF1*, *MMP9*), as well as neutrophil extracellular traps (NETs) formation (*PADI4*).

### 2.4. PSE: Dysregulation of ECM Repair

#### 2.4.1. Systemic Venous Endothelial Cells Were Upregulated in PSE, with Pronounced Transcriptional Perturbations

**Figure 5:**
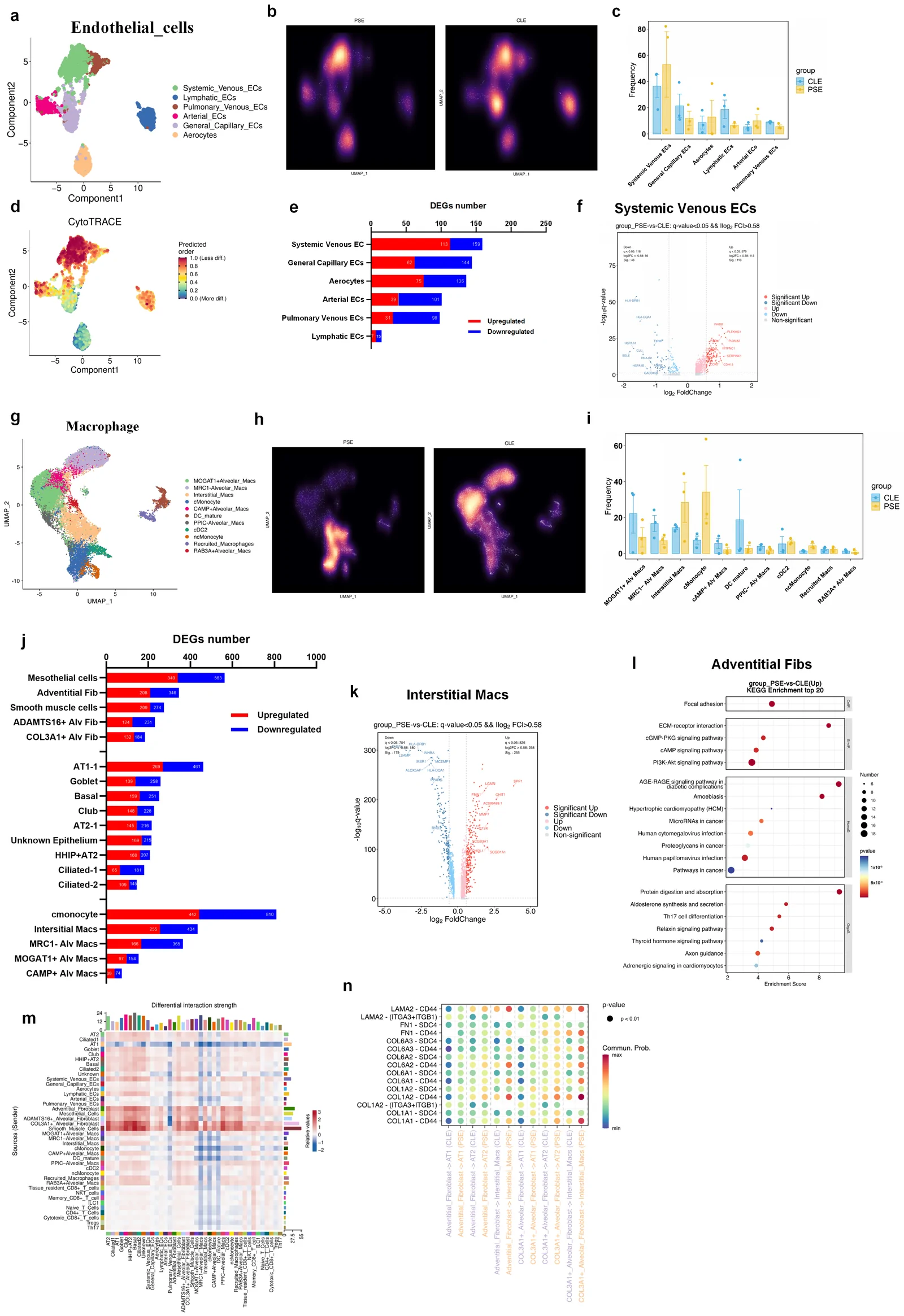
Distinct abundance and transcriptional traits of endothelial cells, interstitial macrophages and adventitial fibroblasts in PSE. (**a**) UMAP plot shows six distinct subclusters of endothelial cells. (**b**,**c**) Density plot and bar plot show the relative proportions of endothelial cell subtypes in CLE and PSE, respectively. The brighter areas in (**b**) indicate a higher number of cells. (**d**) UMAP plot shows the differentiation potential of distinct endothelial subclusters calculated by CytoTRACE analysis. (**e**) Bar plot shows the numbers of upregulated and downregulated DEGs in each endothelial cell subtype between CLE and PSE. (**f**) Volcano plot shows the DEGs of systemic venous endothelial cells between PSE and CLE. (**g**) UMAP plot shows eleven distinct subclusters of macrophages. (**h**,**i**) Density plot and bar plot show the relative proportions of macrophage subtypes in CLE and PSE, respectively. The brighter areas in (**h**) indicate a higher number of cells. (**j**) Bar plot shows the number of upregulated and downregulated DEGs in each mesenchymal, epithelial and macrophage subtype between PSE and CLE. (**k**) Volcano plot shows DEGs of interstitial macrophages between PSE and CLE. (**l**) Dot plot shows enriched KEGG pathways of adventitial fibroblasts between PSE and CLE. (**m**) Heatmap shows differences in inferred cell–cell interaction strength among epithelial cells, endothelial cells, macrophages, mesenchymal cells, and T cells between PSE and CLE. (**n**) Dot plot shows inferred cell–cell communication probabilities between ligand–receptor pairs across major cell types from PSE and CLE. Abbreviations: CLE, centrilobular emphysema; PSE, paraseptal emphysema; UMAP, uniform manifold approximation and projection; KEGG, Kyoto Encyclopedia of Genes and Genomes; DEGs, differentially expressed genes.

Endothelial cells (ECs) were further subjected to dimensionality reduction and unsupervised clustering, resulting in six distinct clusters (Figure 5a). Based on their expression profiles, these clusters were annotated as Systemic venous ECs, Lymphatic ECs, Pulmonary Venous ECs, Arterial ECs, General Capillary ECs and Aerocytes (Figure 5a). Comparative analysis showed that the proportions of Systemic venous ECs were higher in PSE compared with CLE (Figure 5b,c). Furthermore, CytoTRACE analyses suggested that, compared with other ECs, Systemic venous ECs displayed the lowest differentiation state and retained differentiation potential, while Aerocytes were at the highest differentiation state (Figure 5a–d). DEG analysis demonstrated that genes upregulated in PSE compared with CLE were predominantly concentrated in Systemic venous ECs, including *INHBB*, *SERPINE1*, *COL1A1*, *PXDN*, *CDH13*, and *CCN2* (Figure 5e,f). In addition, the top 20 DEGs of all subclusters and significantly enriched pathways (PSE vs. CLE) are listed in Tables S7 and S8.

#### 2.4.2. Increased Proportion of Interstitial Macrophages in PSE, Along with Enhanced Interactions with Fibroblasts

Comparative analysis showed that the proportions of interstitial macrophages and cMonocytes were higher in PSE compared with CLE, whereas the proportions of the top three alveolar macrophages were decreased in PSE (Figure 5g–i). DEG analysis demonstrated that mesothelial cells, adventitial fibroblasts, AT1, cMonocytes, and interstitial macrophages exhibited the most pronounced transcriptional perturbations in PSE (Figure 5j). Volcano plots showed that *SPP1*, *LGMN*, *MFN1*, *CHIT1*, *MMP7*, *CTSK* and *CHI3L1* were significantly increased in interstitial macrophages in PSE compared with CLE (Figure 5k). Enrichment analyses suggested that, compared with CLE, pathways including ECM–receptor interaction, focal adhesion, cyclic guanosine monophosphate–protein kinase G (cGMP-PKG) signaling pathway, cyclic adenosine monophosphate (cAMP) signaling pathway, PI3K-Akt signaling pathway and protein digestion and absorption were upregulated in adventitial fibroblasts from PSE (Figure 5l).

Cell–cell communication analyses suggested that, compared with CLE, the interactions of adventitial fibroblasts and *COL3A1*+ alveolar fibroblasts with epithelial cells and macrophages were significantly enhanced in PSE (Figure 5m). ECM–receptor interactions were significantly upregulated in PSE, including LAMA2-CD44, COL6A2-CD44, COL1A2-CD44, and COL1A1-CD44 pairs (Figure 5n). In addition, the top five cell–cell communication ligand–receptor pairs in each of the three different emphysema types are listed in Table S9. The number of interactions and interaction strength in three different emphysema types are presented in Figure S6.

### 2.5. Sensitivity Analyses

To assess the robustness and disease relevance of the subtype-associated molecular features identified in the single-cell analyses, we performed two complementary sensitivity analyses: a patient-level pseudobulk differential expression analysis and an external validation using healthy lung samples from GSE196638.

In the pseudobulk analysis, the overall biological features identified in CLE and PSE were partially supported at the patient level. In CLE, macrophage subpopulations showed enrichment of immune- and inflammation-related biological processes, including antigen processing and presentation, immune cell recruitment, inflammatory signaling, and tissue remodeling (Table S10). In PSE, several genes, including *SPP1*, *CHIT1*, *MMP7*, and *CTSK*, remained significantly differentially expressed, and pathway enrichment showed substantial concordance with the primary analysis. In particular, ECM–receptor interaction, focal adhesion, PI3K-Akt signaling, cGMP-PKG signaling, cAMP signaling, and protein digestion and absorption remained enriched, further supporting extracellular matrix remodeling as a major molecular feature of PSE (Table S11). These patient-level analyses therefore supported the major biological interpretations of the primary single-cell analyses, whereas the incomplete reproduction of individual gene-level differences highlights the importance of accounting for patient-level variability and the limited statistical power of pseudobulk analyses.

Next, we used healthy lung samples from GSE196638 as an external reference to determine whether subtype-associated molecular features were also evident in disease-versus-healthy comparisons (Tables S12–S17). Compared with healthy controls, CLE showed enrichment of biological processes related to bacterial invasion of epithelial cells, regulation of protein localization to cilia, and cellular senescence. Several genes identified in the CLE-versus-PLE comparison, including *LCN2*, *SCGB1A1*, *APOE*, *RND3*, *ADAMTS1*, *PRG4*, and *FMN1*, also showed higher expression in CLE than in healthy controls, providing additional support for ciliated epithelial alterations and senescence-associated features in CLE (Tables S12 and S15).

Compared with healthy controls (Tables S13 and S16), PLE showed increased expression of *FCGR3B*, consistent with the neutrophil-associated signatures identified in the primary analysis. *PLA2G2A*, which was predominantly expressed in mesothelial cells, was also increased in PLE, supporting the involvement of mesothelial cells in this subtype. In addition, *EGR2*, a transcription factor associated with NKT-cell differentiation and maturation, was upregulated in PLE, consistent with the increased abundance of NKT cells observed in the single-cell analysis. Although pathway-level concordance was less extensive for PLE than for the other subtypes, these findings provide additional evidence for neutrophil-, mesothelial cell-, and NKT cell-associated alterations in PLE.

Among the three emphysema subtypes, PSE showed the greatest concordance with the primary subtype-specific findings (Tables S14 and S17). Compared with healthy controls, PSE exhibited enrichment of pathways related to TGF-β signaling, integrin signaling, ECM–receptor interaction, focal adhesion, and extracellular matrix organization, further supporting extracellular matrix remodeling as a defining molecular feature of PSE. Consistently, several genes highlighted in the primary analyses, including *CHIT1*, *MMP7*, *CTSK*, *SCGB3A1*, and *SCGB1A1*, were also upregulated in PSE relative to healthy controls. In contrast, several immune-related pathways, including antigen processing and presentation, T helper type 1 (Th1)/T helper type 2 (Th2) cells and T helper type 17 (Th17) cells cell differentiation, Nuclear factor kappa B (NF-κB) signaling, Toll-like receptor signaling, and neutrophil activation, were reduced in PSE compared with healthy controls, suggesting that extracellular matrix remodeling rather than inflammatory activation represents the predominant molecular feature of PSE.

## 3. Discussion

To our knowledge, this study is the first comprehensive scRNA sequencing analysis comparing distinct subtypes of emphysema. We systematically characterized the cellular composition, transcriptional signatures, and intercellular communication networks across CLE, PLE, and PSE, providing new insights into the cellular and molecular heterogeneity underlying emphysema pathogenesis.

Increasing evidence indicates that COPD is a heterogeneous disease composed of distinct inflammatory and cellular phenotypes rather than a single pathological entity. A recent multilevel immunoprofiling study that integrated flow cytometry, cytokine profiling, single-cell transcriptomics, and spatial transcriptomics identified an inflammation-associated COPD subgroup characterized by increased antigen-presenting cells, CD8+ T cells, and circulating inflammatory mediators, which was associated with greater emphysema severity independently of Global Initiative for Chronic Obstructive Lung Disease (GOLD) stage. These findings highlight the importance of inflammatory programs in shaping COPD heterogeneity [19].

Consistent with this concept, our findings suggest that CLE represents an inflammation-enriched emphysema subtype with coordinated alterations across epithelial, immune, and stromal compartments. Current evidence suggests that CLE primarily originates in the respiratory bronchioles [18]. In our study, we observed that CLE exhibited an increased proportion of ciliated cells accompanied by upregulated expression of inflammatory genes, in comparison with PLE. Previous studies have demonstrated that ciliated cells contribute to the clearance of pathogens and particulates through coordinated ciliary beating [20,21]. Our enrichment analysis further indicated that ciliated cells in CLE were associated with pathways involved in neutrophil degranulation, anti-apoptotic processes, and antigen processing and presentation compared with PLE. Changes in the number and function of ciliated cells imply that different subtypes of emphysema may have distinct pathological differences in areas above the level of the respiratory bronchioles. Although these findings represent relative differences between emphysema subtypes rather than comparisons with healthy lungs, they are consistent with previous evidence suggesting that ciliated cell dysfunction, potentially driven by recurrent infection or cigarette smoke exposure, may be more closely associated with the pathogenesis of CLE.

Macrophages also exhibited marked alterations in CLE. Although GSVA analyses revealed functional heterogeneity among the five major macrophage subclusters, the comparison between CLE and PLE identified a shared set of significantly upregulated DEGs, including *SPP1*, *CCL18*, *SCGB1A1*, and *NAMPT*. Similarly, a previous single-cell RNA sequencing analysis of peripheral blood mononuclear cells from patients with COPD demonstrated an increased proportion of monocyte/macrophages and identified multiple inflammation-related DEGs, including *CXCL8*, *IL1B* and *CD83* [22]. Together, these findings support the notion that macrophage activation is a prominent feature of COPD and may contribute to disease heterogeneity across different tissue compartments. These transcriptional differences suggest that macrophages in CLE may exhibit greater involvement in inflammatory dysregulation, immune cell recruitment, metabolic reprogramming, and tissue remodeling relative to those in PLE [23,24,25,26,27]. In addition, cell–cell communication analyses demonstrated that macrophages’ interactions with epithelial cells and fibroblasts were markedly enhanced in CLE compared with PLE. Among the ligand–receptor pairs, the MIF-(CD74 + CXCR4) axis exhibited the most prominent differences. Similarly, a previous study also demonstrated that MIF binding to CD74 could promote the migration of glucocorticoid-sensitive fibroblasts, facilitating tissue repair [28].

Our analyses also revealed a significant enrichment of adventitial fibroblasts in CLE compared with PLE, which suggested that adventitial fibroblasts might act as key effector cells in CLE pathogenesis. Adventitial fibroblasts are recognized as active regulators of vascular and perivascular inflammation [29], capable of secreting cytokines and chemokines that sustain immune cell recruitment [30,31,32]. Functionally, genes exhibiting higher expression in CLE than in PLE are mainly associated with inflammation, cellular senescence, and TGF-β signaling. Notably, the enrichment of senescence-related pathways and TGF-β pathways both indicated that these fibroblasts might acquire a senescence-associated secretory phenotype, thereby contributing to ECM remodeling [33,34]. These subtype-specific transcriptional features might reflect a dysregulated injury–repair axis in CLE, wherein persistent activation and senescence of adventitial fibroblasts favor maladaptive tissue remodeling over effective resolution of lung injury. Taken together with previous evidence on ciliated cell biology, these findings raise the possibility that impaired ciliated cell barrier function may act as an inflammatory trigger in CLE. Through interactions among ciliated cells, macrophages, and fibroblasts, airway-derived inflammatory signaling may contribute to fibroblast senescence and impaired tissue repair.

PLE is another clinically most relevant variant of emphysema, which is thought to be related to Alpha-1 Antitrypsin Deficiency (AATD) [10]. AATD is a genetic disorder that leads to reduced levels of alpha-1 antitrypsin in the blood, which functions to inhibit neutrophil elastase. In our study, we observed that genes associated with neutrophil activation and NETs were upregulated in PLE relative to CLE. A previous study showed that NETs could induce airway inflammation via cyclic GMP-AMP synthase (cGAS)/Toll-like receptor 9 (TLR9)-dependent NF-κB activation, suggesting that NETs might also contribute to the inflammatory microenvironment in PLE [35]. Another study in Porcine pancreatic elastase (PPE)-induced emphysema models identified a lung-resident, pro-inflammatory Siglec-F+ neutrophil subset that promoted airway inflammation [36]. Given that PLE shares pathological features with elastase-induced emphysema modeled by PPE administration, it is possible that specific neutrophils may also contribute to the inflammatory microenvironment in PLE, which warrants further investigation in future studies.

In addition to neutrophils, the proportion of NKT cells and ILC1 cells in PLE was elevated relative to CLE. Some studies suggest that NKT cells can exert direct cytotoxic effects on pulmonary epithelial cells through the release of perforin and granzymes [37,38,39]. In our study, we found that NKT cells in PLE displayed an enrichment of granzyme-mediated programmed cell death, which is consistent with previous studies. ILCs are specialized leukocytes of lymphoid origin that are divided into three major helper-like subsets, ILC1, ILC2 and ILC3. Recent studies have suggested that ILC1s play an important role in COPD. Blomme et al. revealed that an increased frequency of ILC1s in the peripheral blood of COPD patients is correlated with disease severity and increased exacerbation risk [40]. Additionally, ILC1s were found to be expanded in the lung of severe COPD patients [41]. ILC1s can produce Interferon-γ (IFN-γ), which stimulates alveolar macrophages to produce elastases and nitric oxide, thereby leading to emphysema [42,43,44]. Similarly, ILC1s in PLE exhibited greater enrichment of cytolytic and degranulation pathways than those in CLE, together with immune effector functions related to leukocyte, neutrophil, and myeloid activation. These observations suggest that ILC1s may drive lung tissue damage through both direct cytotoxicity and amplification of innate inflammatory responses.

Our analysis also revealed a marked expansion of mesothelial cells in PLE. CytoTRACE analysis indicated the lowest differentiation status of mesothelial cells among mesenchymal cells, and enrichment analysis demonstrated significant upregulation of ECM-related pathways in PLE mesothelial cells compared with CLE. Mesothelial cells have not yet been systematically investigated in emphysema. A previous study has shown that mesothelial cells can differentiate into activated fibroblasts and promote pulmonary fibrosis [45]. Taken together, these findings suggest that mesothelial cells in PLE may function as dynamic regulators of the extracellular matrix. Through their progenitor-like properties and enhanced ECM-related activity, these cells may contribute to aberrant matrix remodeling and tissue architecture disruption in PLE. Thus, compared with CLE, PLE exhibited a mixed phenotype, involving both dysregulated innate immune responses—particularly driven by neutrophils, NKT cells, and ILC1s—and imbalance in ECM homeostasis of mesothelial cells.

As compared with CLE, PSE exhibited a relative enrichment of systemic venous endothelial cells, which also displayed the largest number of DEGs and retained differentiation potential. The endothelium of the systemic circulation in the lung primarily consists of bronchial venous endothelium, which is predominantly located in the subpleural regions and within the interlobular septa [46]. The observed upregulation of systemic venous endothelial cells may be attributable to the sampling location. Genes showing higher expression in systemic venous endothelial cells from PSE relative to CLE include *INHBB*, *SERPINE1*, *COL1A1*, *PXDN*, *CDH13*, and *CCN2*. Interestingly, some of these genes (*COL1A1*, *CCN2* and *SERPINE1*) are key mediators of collagen deposition and fibroblast activation, contributing to lung fibrosis [47,48]. Meanwhile, some studies reported upregulation of *INHBB* and *CDH13*, pointing toward activation of TGF-β-related signaling and endothelial remodeling, respectively [49,50,51]. Collectively, these transcriptional features suggest that systemic venous endothelial cells in PSE may exhibit a more profibrotic and remodeling-prone phenotype than those in CLE, potentially contributing to disease-specific vascular and matrix alterations.

We also noticed an increased proportion of interstitial macrophages in PSE, with higher expression of genes including *SPP1*, *LGMN*, *MFN1*, *CHIT1*, *MMP7*, *CTSK*, and *CHI3L1* relative to CLE, which suggests an important role of these cells in tissue remodeling and fibrosis in PSE [52,53,54]. Similarly, interstitial Lyve-1+ macrophages have been reported to regulate the amount, composition, and organization of the pulmonary collagen network at steady state, thereby influencing COPD development [55]. Our study also revealed enhanced ECM–receptor interactions between interstitial macrophages and fibroblasts in PSE relative to CLE, including LAMA2-CD44, COL6A2-CD44, and COL1A2-CD44. These ligand–receptor interactions showed greater predicted interaction strength in PSE relative to CLE and may facilitate fibroblast activation and subsequent ECM deposition [56,57]. Taken together, these subtype-specific molecular features suggest that PSE exhibits more prominent ECM remodeling and profibrotic characteristics than CLE, recapitulating several features of fibrotic lung diseases.

Importantly, the robustness of these subtype-specific findings was further evaluated using patient-level pseudobulk analyses and an external healthy reference dataset. The pseudobulk analyses largely supported the major biological programs identified in the single-cell analyses, particularly the immune- and inflammation-related alterations in CLE, and the extracellular matrix remodeling program in PSE. Notably, several PSE-associated genes, including *SPP1*, *CHIT1*, *MMP7*, and *CTSK*, remained significantly differentially expressed at the patient level, and ECM-related pathways showed consistent enrichment. These findings suggest that the prominent remodeling phenotype observed in PSE is not solely driven by cell-level variability. The external comparison with healthy lung samples from GSE196638 further demonstrated that several subtype-associated features were also evident relative to non-emphysematous lung tissue. In particular, CLE showed features related to ciliated epithelial dysfunction and cellular senescence, PLE exhibited neutrophil-, mesothelial cell-, and NKT cell-associated alterations, and PSE showed the strongest concordance with the extracellular matrix and profibrotic programs identified in the primary analyses. Although not all individual genes or pathways were consistently reproduced across different analytical approaches, the overall concordance supports the robustness of the major biological programs identified in each emphysema subtype.

Our study has several limitations. First, the relatively small number of patients in each emphysema subtype limits the statistical power of the study and may reduce the reproducibility of subtype-specific differential expression and cellular composition analyses. This limitation suggests that these observed cellular and transcriptional differences may represent exploratory findings rather than definitive disease mechanisms. Accordingly, these findings should be interpreted cautiously and should not be considered as evidence of causality. The patient-level pseudobulk sensitivity analyses provided complementary support for several major biological findings, but individual gene-level differences were not uniformly reproduced, further highlighting the influence of patient-level variability and limited statistical power. Larger independent cohorts with balanced numbers of patients across emphysema subtypes will be required to confirm the reproducibility and biological significance of these subtype-specific findings. Second, considering the limited sensitivity of scRNA-seq for detecting rare populations, we restricted our analysis to abundant cell subsets and did not analyze rare subclusters, such as club cells and basal cells. Third, this scRNA-seq study did not directly measure protein abundance, post-translational modifications, or spatial location information. Owing to the lack of surface protein information, we were unable to further subtype CD4+ T cells [58]. Another limitation is the absence of matched healthy lung controls. Our analyses identify relative cellular and molecular differences between emphysema subtypes, rather than absolute alterations relative to the normal lung. Consequently, the observed enrichment of specific pathways in one subtype does not necessarily reflect definitive activation relative to healthy tissue, but rather captures subtype-specific differences. To partially address this limitation, we incorporated healthy lung samples from GSE196638 as an external reference in a sensitivity analysis. This analysis provided additional evidence that several key subtype-associated molecular features were also detectable relative to healthy lung tissue, although the concordance was not complete across all genes and pathways. Because the external controls were derived from an independent dataset, differences in cohort characteristics, sample processing, and technical platforms may have introduced potential batch effects and limited the comparability between datasets. Therefore, these external comparisons should be interpreted as complementary evidence rather than a substitute for matched healthy controls. Future studies incorporating well-matched healthy lung samples and larger independent cohorts will be important to further validate the subtype-specific molecular programs identified in this study.

## 4. Materials and Methods

### 4.1. Patient Recruitment and Sample Preparation

We recruited patients diagnosed with emphysema who underwent lung resection surgery for lung cancer. Demographic characteristics and pulmonary function parameters were recorded. Emphysema subtypes were determined by chest computed tomography (CT) and assessed by two independent pulmonologists. Lung tissue samples were obtained from regions of emphysematous lung that were free of tumor infiltration. A total of nine patients were included in this study, with three patients representing each emphysema subtype (CLE, PLE, and PSE). Representative CT images and the subsequent workflow are illustrated in Figure 1a.

### 4.2. Single-Cell RNA Sequencing and Data Preprocessing

The sequencing was provided by OE Biotech Co., Ltd. (Shanghai, China). The 10x Genomics Chromium Controller platform with Chromium Next GEM Single Cell 3′ Reagent Kits v3.1 was used to assemble 3′ gene expression libraries. Sequencing was performed on an Illumina NovaSeq 6000 instrument (Illumina, Inc., San Diego, CA, USA). Raw sequencing data were in FASTQ format and processed using the official 10x Genomics software, Cell Ranger (version 9.0.0), for quality control and alignment to the human reference genome (GRCh38).

Following preliminary quality control with Cell Ranger, further processing was conducted using the Seurat R package (version 4.0.0) [59]. Cells were filtered based on the following criteria: gene count > 200, Unique Molecular Identifier (UMI) count > 1000, log10GenesPerUMI > 0.7, mitochondrial UMI proportion < 15%, and hemoglobin gene proportion < 5%. Doublet cells were removed using DoubletFinder (version 2.0.3) [60]. High-quality cells passing these criteria were normalized using the NormalizeData function in Seurat. Highly variable genes (HVGs) were identified with the FindVariableGenes function (mean.function = FastExpMean, dispersion.function = FastLogVMR), and the top 2000 HVGs were selected for downstream analyses. PCA was performed on the HVGs, and UAMP was used for two-dimensional visualization of the data.

### 4.3. Cell Type Annotation and Top 10 Marker Gene Identification

Cell clusters were annotated using SingleR (version 1.4.1), which calculates correlation between the expression profiles of sample cells and reference datasets, assigning the cell clusters with the highest correlation as the final annotation [61]. The annotation results were then cross-validated and supplemented based on known lineage markers for various lung cell types reported in the published literature [17]. Clusters with ambiguous expression profiles could not be reliably annotated and were therefore excluded from further analysis. Following the identification of cell types, subcluster annotation was further conducted based on reference to known lineage markers for lung cell subtypes reported in a previous study [17]. Subclusters that either lacked clear lineage gene expression or had co-expressed marker genes from two distinct clusters were excluded from downstream analyses.

Top 10 marker genes for each cell cluster were identified using the FindAllMarkers function (test.use = presto). The identified top 10 marker genes were visualized using the VlnPlot and FeaturePlot functions. The top 10 marker genes of each subcluster are listed in Supplementary Tables.

### 4.4. Differentially Expressed Genes and Enrichment Analysies

Differentially expressed genes (DEGs) between groups were identified using the FindMarkers function (test.use = presto), with thresholds set at *p*-value < 0.05 and fold change > 1.5. Significant DEGs were subsequently subjected to Gene Ontology (GO) and Kyoto Encyclopedia of Genes and Genomes (KEGG) enrichment analyses using the hypergeometric test implemented in the clusterProfiler R package(version 4.8.3). Visualization of enrichment results was performed using ggplot2 (version 3.4.3) and enrichplot (version 1.20.1). Similarly, we performed DEG analyses between groups within each cell subtype and conducted KEGG and GO enrichment analyses using the significant DEGs. Detailed top 20 DEGs and enrichment analyses are reported in Supplementary Tables.

### 4.5. Pseudotime Analysis

The Monocle2 R package (version 2.26.0) was utilized to perform machine learning based on the expression patterns of key genes, and then to model the dynamic developmental changes in various cell subtypes over time [62]. First, genes exhibiting high variability in expression across cells were selected. Dimensionality reduction was then performed based on their expression profiles, followed by construction of a minimum spanning tree (MST). The longest path along this MST was subsequently identified to represent the differentiation trajectory of cells with similar transcriptional characteristics. In addition, the CytoTRACE R package (version 0.3.3) was applied to further infer the relative differentiation states and developmental direction of individual cells based on gene expression entropy [63].

### 4.6. GSVA

A rank-based, Kolmogorov–Smirnov-like calculation was performed on gene sets derived from the KEGG and GO:BP databases, transforming the expression matrix of each cell into a pathway enrichment score (ES) matrix [64]. This yielded GSVA enrichment scores for each pathway in each cell. Subsequently, the limma package (version 3.62.2) was used to identify pathways exhibiting significant differences between cell subtypes [65], allowing us to assess the relative enrichment of different pathways across distinct cellular subpopulations. GSVA features of each subcluster are listed in the Supplementary Tables.

### 4.7. Cell–Cell Communication

Cell–cell communication analysis was performed using the R package CellChat V2 (version 2.1.2) [66,67] to investigate intercellular signaling among major cell lineages in different emphysema subtypes. Specifically, we focused on epithelial cell subtypes, endothelial cell subtypes, mesenchymal cell subtypes, macrophage subtypes, and T cell subtypes. For each group, CellChat was used to infer the number and strength of potential ligand–receptor interactions. Comparative analyses were conducted to identify differences in cell–cell communication patterns between CLE and PLE, as well as between PSE and CLE, highlighting subtype-specific alterations in intercellular signaling networks.

### 4.8. Sensitivity Analyses

To assess the robustness of the main findings, two additional sensitivity analyses were performed. First, pseudobulk differential expression analysis was conducted to validate the principal differential expression findings identified by single-cell-level analysis. For each cell subtype, gene expression counts were aggregated across cells from the same patient to generate patient-level pseudobulk profiles, with each patient treated as an independent biological replicate. Differential expression analysis was then performed between the corresponding study groups using the aggregated patient-level expression profiles. The resulting differentially expressed genes were compared with those identified in the primary single-cell analyses.

Second, an external healthy control dataset from GSE196638 was incorporated as an independent reference. Healthy control samples were processed using the same analytical framework and were compared with emphysema samples from our cohort. For each emphysema subtype, differential expression analyses were performed between the subtype and the external healthy control group to determine whether the molecular features identified in the primary analyses were consistently observed in disease-versus-healthy comparisons.

### 4.9. Software and Statistical Analysis

For the demographic and clinical characteristics of nine participants, continuous variables were presented as mean ± standard deviation, and categorical variables were summarized as counts and percentages. For comparisons among the three groups, the Kruskal–Wallis test was used for continuous variables, while Fisher’s exact test was applied for categorical variables as appropriate. A two-sided *p*-value < 0.05 was considered statistically significant. All analyses were performed using R software (version 4.4.2).

## 5. Conclusions

Our study suggests that emphysema subtypes are mechanistically heterogeneous, with CLE being predominantly driven by airway-originated inflammation, PSE displaying a remodeling- and fibrosis-like phenotype, and PLE representing an intermediate state of innate immune activation and matrix dysregulation.

## Figures and Tables

**Table 1:** Demographic and clinical characteristics of 9 participants.

		CLE (*n* = 3)	PLE (*n* = 3)	PSE (*n* = 3)	*p*-Value
Age		65.33 ± 5.51	68.67 ± 6.43	72.67 ± 5.69	0.315
Sex	Male	3 (100%)	3 (100%)	3 (100%)	1
Height (m)		1.65 ± 0.07	1.65 ± 0.15	1.70 ± 0.10	0.528
Weight (kg)		53.0 ± 3.61	59.33 ± 14.01	59.67 ± 4.51	0.413
BMI (kg/m^2^)		19.41 ± 1.23	21.72 ± 3.12	20.75 ± 3.23	0.561
Smoking status	Yes	3 (100%)	3 (100%)	3 (100%)	1
Years of smoking		30.33 ± 20.82	40.00 ± 0.00	43.33 ± 5.77	0.740
Cigarettes per day		26.67 ± 11.54	29.17 ± 11.27	20.00 ± 0.00	0.695
Tuberculosis	Yes	0 (0%)	0 (0%)	0 (0%)	1
History of previous lung surgery	Yes	0 (0%)	0 (0%)	0 (0%)	1
	No	3 (100%)	3 (100%)	3 (100%)	
FEV1%		52.80 ± 31.68	52.80 ± 23.48	79.63 ± 42.95	0.430
FEV1/FVC		82.47 ± 15.00	75.06 ± 8.91	84.01 ± 15.76	0.733
DLCO%		60.87 ± 26.74	51.37 ± 13.69	65.27 ± 31.52	0.733

Abbreviations: FEV1%, forced expiratory volume in one second, percent predicted; FEV1, forced expiratory volume in one second; FVC, forced vital capacity; DLCO%, diffusing capacity of the lung for carbon monoxide, percent predicted.

## Data Availability

The raw sequence data reported in this paper have been deposited in the Genome Sequence Archive (Genomics, Proteomics & Bioinformatics 2025) in the National Genomics Data Center (Nucleic Acids Res 2026), China National Center for Bioinformation/Beijing Institute of Genomics, Chinese Academy of Sciences (GSA: HRA018550) that are publicly accessible at https://ngdc.cncb.ac.cn/gsa (accessed on 23 August 2026).

## Supplementary Materials

The following supporting information can be downloaded at: https://www.mdpi.com/article/10.3390/ijms27177807/s1.

## Abbreviations

CLE	centrilobular emphysema
PLE	panlobular emphysema
PSE	paraseptal emphysema
ECM	extracellular matrix
COPD	chronic obstructive pulmonary disease
scRNA	Single-cell RNA
CT	computed tomography
HVGs	Highly variable genes
PCA	Principal component analysis
UAMP	uniform manifold approximation and projection
DEGs	Differentially expressed genes
GO	Gene Ontology
KEGG	Kyoto Encyclopedia of Genes and Genomes
MST	minimum spanning tree
GSVA	Gene set variation analysis
ES	enrichment score
ILC1	Innate lymphoid cells 1
NETs	neutrophil extracellular traps
ECs	Endothelial cells
AATD	Alpha-1 Antitrypsin Deficiency
