# Supplementary material for: A Single-Cell Transcriptional Landscape of Emphysema: Heterogeneous Cellular Dynamics in Centrilobular, Panlobular, and Paraseptal Subtypes
Source: Int J Mol Sci. 2026 Aug 31;27(17):7807. doi: 10.3390/ijms27177807 (PMC13566781; doi:10.3390/ijms27177807)
Supplement: Supplementary file 1 [file ijms-27-07807-s001.zip › supplement Figure legend v26-5-4.pdf]

sFigure1. Quality control of single-cell RNA-seq

(a) Violin plot showed the distribution of numbers of RNA for each participant after quality control filtering; (b) Violin plot showed the distribution of number of features for each participant after quality control filtering; (c) Violin plot showed the distribution of percentage of hemoglobin gene expression for each participant after quality control filtering; (d) Violin plot showed the distribution of percentage of mitochondrial gene expression for each participant following quality control.

sFigure 2. Distribution and correlation of each cellular cluster

(a) UMAP embedded of all cells colored according to emphysema subtypes (PLE, CLE, and PSE); (b) UMAP embedded of all cells colored according to each participant. (c) Stacked bar plot showed the relative proportions of each unsupervised cluster across individual participants; (d) Heatmap showed the correlation between different cell clusters. Color intensity reflects the strength of correlation, with red indicating higher correlation and blue indicating lower correlation.

sFigure 3. Distribution of cellular subclusters in three groups

(a) Stacked bar plot showed the relative proportions of each epithelial cells subtype across emphysema subtypes; (b) Stacked bar plot showed the relative proportions of each endothelial cells subtype across emphysema subtypes; (c) Stacked bar plot showed the relative proportions of each T cells subtype across emphysema subtypes; (d) Stacked bar plot showed the relative proportions of each Macrophages subtype across emphysema subtypes; (e) Stacked bar plot showed the relative proportions of each B cells subtype across emphysema subtypes

sFigure 4. Pseudotime analysis of cellular subclusters in three groups

(a) Trajectory plot showed the inferred pseudotime ordering of epithelial cells; (b) Branched heatmap showed dynamically expressed genes along the pseudotime trajectory of epithelial cells. The x-axis represents genes. The gray bar at the top indicates the branch point of the trajectory, while the left (red) and right (blue) bars represent distinct developmental stages following the branch. Color intensity in the heatmap ranges from blue to red, indicating low to high gene expression levels. Genes are grouped into four modules (blue, green, purple, and red) based on similar expression patterns along the developmental trajectory, highlighting distinct temporal gene expression programs; (c) Bar plot showed Gene Ontology (GO) enrichment analysis for the top 30 genes in Module 3 identified from pseudotime clustering in epithelial cells; (d) Line plot showed the average expression dynamics of genes within each of the four modules along the pseudotime trajectory of epithelial cells. The x-axis represents pseudotime progressing from early to late stages, and the y-axis indicates average gene expression levels. Solid and dashed lines represent different cell states following trajectory branching; (e) Pie charts showed the proportion of epithelial cells in different states within each emphysema subtype (PLE, CLE, and PSE); (f) Trajectory plot showed the inferred pseudotime ordering of endothelial cells; (g) Pie charts showed the proportion of endothelial cells in different states within each emphysema subtype (PLE, CLE, and PSE); (g) Trajectory plot showed the inferred pseudotime ordering of mesenchymal cells; (i) Pie charts showed the proportion of mesenchymal cells in different states within each emphysema subtype (PLE, CLE,

and PSE); (j) Trajectory plot showed the inferred pseudotime ordering of CD4+ T cells. The results are shown separately for each emphysema subtype (PLE, CLE, and PSE), as well as in a combined analysis including all subtypes; (k) Trajectory plot showed the inferred pseudotime ordering of CD8+ T cells. The results are shown separately for each emphysema subtype (PLE, CLE, and PSE), as well as in a combined analysis including all subtypes.

sFigure 5. Pseudotime analysis of macrophage subclusters in three groups

(a) Trajectory plot showed the inferred pseudotime ordering of macrophages. The results are shown separately for each emphysema subtype (PLE, CLE, and PSE); (b) Trajectory plot showed the inferred pseudotime ordering of macrophages. The results are shown separately for each macrophage subtype.

sFigure 6. Interaction number sand strength of cell-cell communication in three groups

(a-b) Heatmap showed differences in inferred cell–cell interaction numbers and strength among epithelial cells, endothelial cells, macrophages, mesenchymal cells, and T cells in CLE; (c-d) Heatmap showed differences in inferred cell–cell interaction numbers and strength among epithelial cells, endothelial cells, macrophages, mesenchymal cells, and T cells in PLE; (d-f)Heatmap showed differences in inferred cell–cell interaction numbers and strength among epithelial cells, endothelial cells, macrophages, mesenchymal cells, and T cells in PSE.
